# Environmentally dependent developmental induction as a potential driver of heart evolution

**DOI:** 10.1242/jeb.250920

**Published:** 2026-01-29

**Authors:** Nina Kraus

**Affiliations:** ^1^ Department of Evolutionary Biology, Theoretical Biology Unit, University of Vienna, Vienna 1030, Austria; ^2^ Konrad Lorenz Institute for Evolution and Cognition Research, 3400 Klosterneuburg, Austria

**Keywords:** Evo-devo, Cardiogenesis, Envionmentally induced modifications, Devo-Evo, Theory

## Abstract

Developmental biology and evolutionary theory have traditionally emphasized gene mutations as the primary drivers of new traits, with natural selection shaping the resulting variation. However, recent insights highlight the role of environmental factors during development in shaping trait evolution. In this Commentary, I introduce the ‘environmentally dependent developmental induction’ (EDDI) model, which proposes that phenotypic evolution is driven not only by genetic changes but also by environmentally induced modifications to the core developmental program. Using cardiogenesis as an example, I argue that environmental triggers such as oxygen levels and mechanical forces expand the genotypic toolkit available to heart development, activating new pathways that lead to the emergence of novel cardiac structures. These lineage-specific environmental changes might thus influence the differentiation of cardiac progenitor cells, resulting in modifications to the cardiac building plan. The EDDI model provides a novel explanation for how the basic cardiac plan was expanded during evolution while simultaneously explaining why cardiogenesis is vulnerable to malformations, even in the absence of genetic defects.

## Teaser

**Summary:** This Commentary introduces the ‘environmentally dependent developmental induction’ model, a potential means through which phenotypic evolution is influenced by environmental effects on development.

## Introduction

Gene mutations are often considered the primary source of new phenotypic traits. However, the development of a phenotype arises from a dynamic interplay between gene-regulatory networks, environmental factors and epigenetic influences (epigenetics *sensu* Newman and Müller, 2000: conditional responses of tissues to each other and to external forces). Epigenetics and the environment provide the physico-chemical substrate necessary for development (Brun-Usan et al., 2022; Newman et al., 2006; Newman and Müller, 2000). As embryos develop, they encounter a characteristic sequence of environmental conditions. Thus, development produces the phenotype, and the variation upon which natural selection acts depends not only on the genotype but also on how the genotype interacts with its environment during development. This suggests that phenotypes evolve not only through genetic changes but also in response to environmental factors (Brun-Usan et al., 2022; Crispo, 2007; Emlen and Nijhout, 2000; Levis and Pfennig, 2016; Newman et al., 2006; Waddington, 1940, 1942, 1959; West-Eberhard, 2005).

The classical Baldwin (1896) effect (see Glossary), Levis and Pfennig's (2016) plasticity-first hypothesis (see Glossary) and West-Eberhard's (2005) genetic accommodation model (see Glossary) put the plastic phenotype at the center of their evolutionary theory. They all suggest that a phenotype's initial plastic response to a novel environment involves the overproduction of random variation, some of which may confer adaptive benefits. If the environment consistently evokes this plastic response over evolutionary time, genetic variation accommodating this adaptive phenotype may emerge, leading to the phenotype's heritable stabilization.

The stress-induced evolutionary innovation model of Wagner et al. (2019) describes the initial response to a new environmental stressor not as random but as a protective stress response within an organism's normal reaction range. If this stressor becomes a persistent feature of the species' life cycle, the stress response may become internalized, producing a compensatory phenotype that is independent of the environment. Over time, this is said to lead to a genetic shift in the reaction norm (see Glossary), facilitating the consistent production of the compensatory phenotype (Love and Wagner, 2022; Wagner et al., 2019).

The aforementioned models have different ideas about how a plastic phenotype allows for evolutionary change, but they all assume that genetic assimilation (see Glossary) of the new developmental program is eventually required to consistently evoke similar adaptive phenotypes. Fittingly, findings from: (1) epigenetics (in the now more common use of the word: ‘the study of changes in gene function that are mitotically and/or meiotically heritable and that do not entail a change in DNA sequence’; Wu and Morris, 2001) and (2) the field of developmental origins of health and disease have shown that environmental conditions during development can produce long-lasting, sometimes transgenerational phenotypic changes without altering the DNA sequence (Lacagnina, 2020).

In contrast to models emphasizing the eventual genetic control of adaptive phenotypes, here, I argue that genetic assimilation might not always occur, particularly in cases where environmental perturbations are known to cause developmental defects. I use vertebrate cardiogenesis to demonstrate that key aspects of the phenotype could remain persistently dependent on environmental triggers. This provides an instructive example of sustained phenotypic plasticity without genetic assimilation while also explaining how development can reliably produce consistent phenotypes in the absence of genetic assimilation.

### Environmentally dependent developmental induction (EDDI)

In this Commentary, I propose that the cardiac developmental program was expanded in response to changes in the developmental environments of different vertebrate classes. I posit that changes to the core developmental program do not become genetically assimilated, but still rely on environmental cues to become induced during cardiogenesis. This model challenges the conventional gene-centric view of heart development and explains how the evolutionary history of the heart leaves its newer accessory structures particularly vulnerable to pathogenesis.

To place this view in a theoretical framework, I introduce a model for the expansion of developmental programs (i.e. networks of gene interactions) through plastic responses to environmental changes, the induction of which does not fall under genetic control. In this view, the regulation of the developmental program remains conditional on certain environmental triggers, and thus depends on properties and mechanisms beyond the strictly genetic. I call this model ‘environmentally dependent developmental induction’ (EDDI; see Glossary). I term the process by which environmental changes activate additional circuits ‘ambiogenic induction’ (see Glossary). Rather than being permanently incorporated into a genetic network, these sub-circuits rely on specific, local, extrinsic factors to be activated during development. Thus, EDDI posits that certain environmental changes during development – such as the significant shift in oxygen levels at the onset of placentation or egg deposition and chorioallantoic membrane (CAM) formation (Carroll et al., 2021; Nowak-Sliwinska et al., 2014) – could lead to ambiogenic induction, which potentially serves as a protective stress response within an organism's normal reaction range (Love and Wagner, 2022; Wagner et al., 2019). The resulting stress response could then recruit new regulatory pathways into the core developmental program, leading to new developmental interactions, altered cell-fate decisions and the subsequent emergence of new phenotypes. Evolutionary shifts in developmental timing – such as changes in egg deposition or placentation – would thus have the potential to influence heart phenotypes in developing embryos.

Within the EDDI model, phenotypic changes could become evolutionarily persistent without requiring genetic assimilation by the core developmental program, as long as the developmental environmental sequence remains stable over evolutionary time (Brun-Usan et al., 2022). If the developmental sequence of environmental cues is consistent across generations, it should reliably evoke the same adaptive phenotype without fixing it through genetic mutations (Kuijper and Hoyle, 2015; Minelli, 2016). This does not suggest that genetic mutations are unimportant but rather that they alone cannot fully explain morphological changes. EDDI posits that genetic changes, although necessary, may not be sufficient to produce novel phenotypes without environmental triggers that also change how genes are expressed. For instance, a gradual genetic shift in a cell's response to oxygen deprivation may only reveal a new phenotype when combined with sudden changes in developmental conditions, making the genetic variation subject to natural selection (Loffet et al., 2023; Waddington, 1940).

Within this Commentary, I discuss how the theoretical framework of EDDI might explain: (1) how developmental programs were expanded during the evolution of different heart phenotypes in vertebrates and (2) why the evolutionary history of the heart leaves its phylogenetically youngest structures especially vulnerable to malformations during development, even in the absence of genetic defects. For this, I review ongoing research in cardiogenesis and present testable predictions made using the EDDI framework.

Glossary **Ambiogenic induction**

A process in which highly localized micro-environmental conditions within a specific tissue or cellular niche act as direct triggers for developmental responses.

**Atavism**

The reappearance of a lost character typical of remote ancestors but not seen in the parents or recent ancestors. Features of the atavistic character are its persistence into adult life, its presence in only one or a few individuals within a population and its close resemblance to/shared identity with the same character possessed by all members of an ancestral population (Hall, 1984).

**Baldwin effect**

The idea that behavioral or developmental plasticity can guide evolution by allowing organisms to survive and reproduce until genetic changes stabilize the adaptive trait.

**Carnegie stages**

A standardized series of 23 stages in the embryonic period of normal human development (Flierman et al., 2023).

**Conotruncal heart defects**

A group of congenital heart malformations involving improper development of the heart's outflow tract, affecting the connection between the ventricles and the great arteries (e.g. truncus arteriosus, Tetralogy of Fallot).

**Endothelial-to-mesenchymal transition (EndoMT)**

Endothelial cells are organized in two dimensions and sit on a basal membrane. Mesenchymal cells have no basal layer and can thus be organized three dimensionally. EndoMT refers to the process where endothelial cells lose their endothelial characteristics and transdifferentiate into a mesenchymal phenotype that has a different genetic expression profile and the ability to migrate.

**Environmentally dependent developmental induction (EDDI)**

A model proposed here that encompasses the expansion of an ancestral developmental network through plastic responses to environmental cues. Within this model, newly recruited developmental circuits are not under the genetic control of the initial network but are triggered by ambiogenic induction.

**Genetic assimilation**

‘The process by which a phenotype that is initially induced by an environmental stimulus becomes stably inherited in the absence of the stimulus after a few generations of selection’ (Raju et al., 2023).

**Haecklian explanation**

Ernst Haeckel's recapitulation theory (also termed ‘biogenetic law’) states that ontogeny (development of an individual organism) recapitulates phylogeny (the evolutionary history of the group) – a view that tends to be seen as overly simplistic by most evolutionary biologists.

**Hamburger–Hamilton stage**

A standardized series of 46 stages in normal chick development from egg deposition to hatching (Hamburger and Hamilton, 1951).

**Levis and Pfennig's plasticity-first hypothesis**

This hypothesis proposes that environmentally induced phenotypes can precede and facilitate genetic evolution by exposing cryptic variation to selection.

**Reaction norm**

‘The spectrum of phenotypic variation produced when individuals of the same genotype are exposed to varying environmental conditions’ (Manuck, 2010).

**West-Eberhard's genetic accommodation model**

A model describing how phenotypic variation, whether environmentally or developmentally induced, becomes stabilized or refined through selection acting on its underlying regulatory mechanisms.

List of symbols and abbreviations

CAM: chorioallantoic membrane
CC: cardiac cushion
CHD: congenital heart disease
cNCC: cardiac neural crest cell
EDDI: environmentally dependent developmental induction
EndoMT: endothelial-to-mesenchymal transition
HIF: hypoxia-inducible factor
OFT: outflow tract
SHF: second heart field
VEGF: vascular endothelial growth factor

## A new perspective on developmental gene expression: integrating oxygenation, apoptosis and evolution in cardiogenic development

### Vertebrate heart evolution

**Fig. 1.**
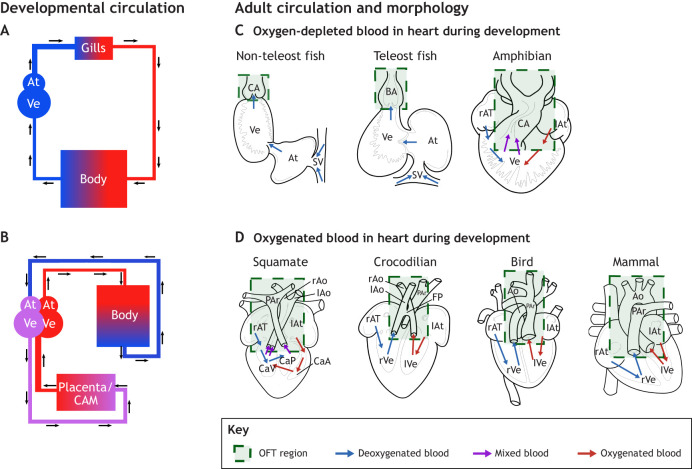
**Modifications to the outflow tract (OFT) of the heart show greatest variability and complexity among different vertebrates.** (A) Schematic depiction of blood flow patterns during development for animals with (B) oxygen-depleted blood in the heart during development. (C) Schematic depiction of blood flow patterns during development for animals with (D) oxygenated blood in the heart during development. Arrows indicate the direction of blood flow; colors correspond to the oxygenation status of blood. Note that all hearts of all classes receiving oxygen-depleted blood during development do not show shortening of the OFT (green boxes) by apoptosis during cardiogenesis. In vertebrate hearts receiving oxygenated blood during development, the OFT shortens and becomes partially incorporated into the ventricle. This completes ventricular separation and leaves each ventricle with its own outflow vessel, leading to a complete, functional double-circulation. (r/l)Ao, (right/left) aorta; (r/l)At, (right/left) atrium; BA, bulbus arteriosus; CA, conus arteriosus; CaA, cavum arteriosum; CAM, chorioallantoic membrane; CaP, cavum pulmonale; CaV, cavum venosum; FP, foramen panizza; OFT, outflow tract; PAr, pulmonary artery; (r/l)Ve, (right/left) ventricle; SV, sinus venosus.

At its core, the vertebrate heart is a muscular pump comprising two functional segments: an inflow tract that receives blood from the venous vascular network and an outflow tract (OFT) that ejects it into the arterial vascular network (Fisher and Burggren, 2007; Simões-Costa et al., 2005). This basic architecture is present during early cardiogenesis in all vertebrates, but the developmental trajectory from this common starting point diverges in phylum- and lineage-specific ways. In jawed fishes, the heart comprises a two-chambered pump with one atrium, one ventricle and an undivided OFT. Amphibians typically have two atria, one ventricle and an undivided OFT containing a septum that might help maintain partial separation between oxygenated and deoxygenated blood (Kraus and Metscher, 2022). Most reptiles also have three chambers, with the ventricle and OFT variably and incompletely divided. In crocodilians, birds and mammals, the heart consists of four chambers forming a functional double circulation (i.e. two completely separated blood circulatory systems) that directs oxygenated blood to the body and deoxygenated blood to the lungs (Fig. 1; Stephenson et al., 2017).

In this Commentary, I focus on the different OFT morphologies. Among vertebrates, modifications to the OFT are phylogenetically the youngest cardiac structures and exhibit considerable adaptability (Fig. 1). This adaptability is essential for regulating blood flow to different gas exchange organs (e.g. lungs, gills, skin; Gittenberger-de Groot et al., 2005; Restivo et al., 2006; Rothenberg et al., 2003). Interestingly, altered cardiac phenotypes in the human OFT often resemble normal phenotypes found in other vertebrates. This raises the possibility that the mechanisms driving macroevolutionary innovations in the OFT may be clinically relevant, potentially contributing to cardiac malformations (Fisher and Burggren, 2007).

### Cardiogenesis and congenital heart malformations

**Fig. 2.**
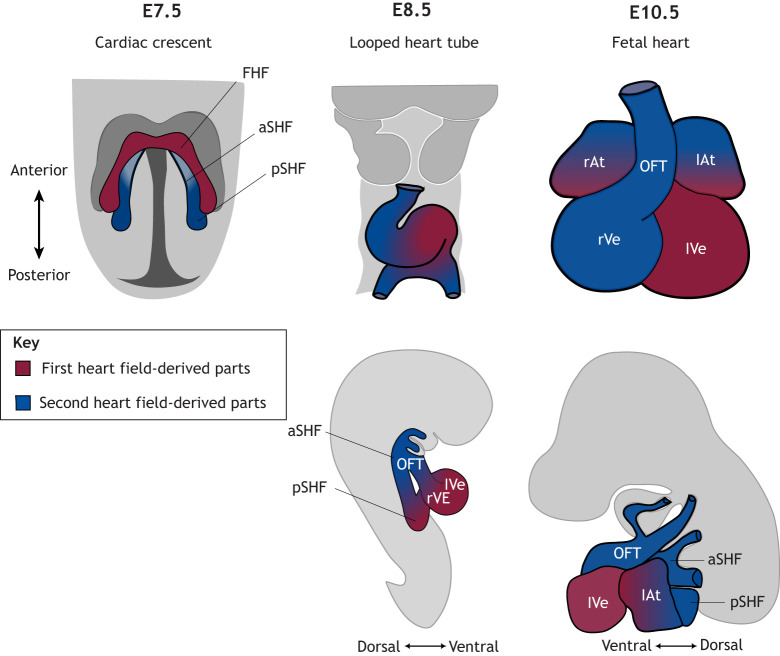
**Schematic illustration of mouse heart development from the cardiac crescent (E7.5) to the fetal heart (E10.5).** The figure highlights the contributions of the first heart field (FHF, red) and second heart field (SHF, blue) to different heart structures. Between E7.5 and E10.5, the SHF progressively provides progenitor cells that differentiate and go on to contribute to various cardiac structures during this developmental window. Drawings based on Hempel and Kühl (2016) (http://creativecommons.org/licenses/by/4.0/) and Kelly (2012). (pr/pl)At, primitive (right/left) atrium; a, anterior; p, posterior; other abbreviations as in Fig. 1.

Cardiac morphogenesis is driven by the specification, proliferation, differentiation and migration of cardiac progenitor cells (Yuan et al., 2017). Specifically, two major populations of cardiac progenitor cells drive early cardiac morphogenesis. The first heart field, composed of early-differentiating cardiac cells, gives rise to part of the left ventricle. Later-differentiating progenitor cells, known as the second heart field (SHF), originate from the pharyngeal mesoderm and contribute to the progressive extension of the heart tube, forming the myocardium of the atria, the ventricle and the inflow and outflow tracts (Fig. 2; Buckingham et al., 2005; Kelly et al., 2014; Kelly and Buckingham, 2002).

**Fig. 3.**
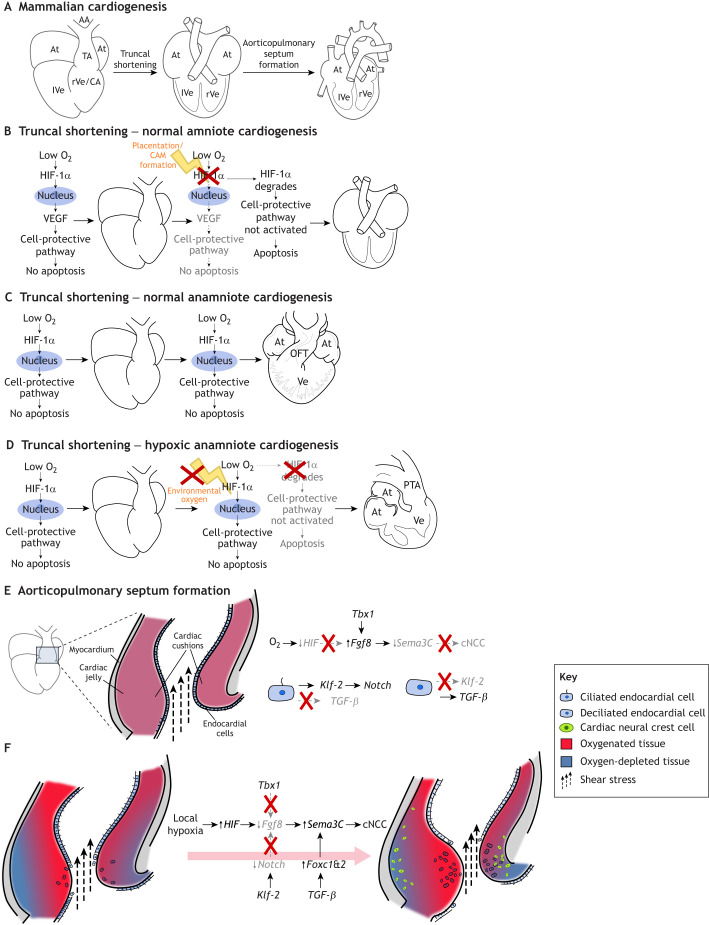
**Mechanistic depiction of crucial steps in OFT development.** (A) Normal mammalian cardiogenesis. As the conus arteriosus portion of the OFT is incorporated into the ventricle, the truncus arteriosus (TA) shortens by apoptosis, coincident with the ingress of oxygenated blood. (B) TA shortening during normal amniote cardiogenesis, where sufficient oxygen availability leads to HIF-1α degradation. This prevents the activation of downstream cell-protective pathways, triggering apoptosis. Apoptosis facilitates OFT remodeling, ultimately reducing the common TA shared by the aorta and pulmonary artery. (C) The normal process in anamniote cardiogenesis, where oxygen-depleted blood flows over the heart. This prevents apoptosis and results in a long TA. (D) Illustration of how the mechanism in amniotes is affected if oxygenation is insufficient to cause HIF degradation. This prevents apoptosis, with a consequent persistent truncus arteriosus (PTA). Completion of the aorticopulmonary (AP) septum requires the migration of cardiac neural crest cells (cNCCs) into the distal OFT. (E) How shear stress regulates gene expression in endocardial cells (ECs). As long as shear stress remains moderate, ECs remain ciliated, but as cardiac cushions continue to swell, some ECs are exposed to higher levels of shear stress, causing their cilium to break. This inhibits *Klf-2* expression, but causes upregulation of *TGF-β*, which is associated with the endothelial-to-mesenchymal transition (EndoMT), where ECs migrate into the cardiac jelly. Together, ciliated and de-ciliated ECs coordinate EndoMT and cNCC migration. In E, OFT tissue is sufficiently oxygenated, so the chemoattractant Sema3C remains downregulated. As depicted in F, when EndoMT progresses sufficiently and the cardiac cushions become thicker and populated with ECs, oxygen diffusion no longer suffices to supply the myocardium, causing local hypoxia and upregulation of *HIF* and its downstream target *Sema3C*, which attracts cNCCs into the developing cardiac cushion, where they contribute to AP septum formation. Abbreviations as in Fig. 1.

**Fig. 4.**
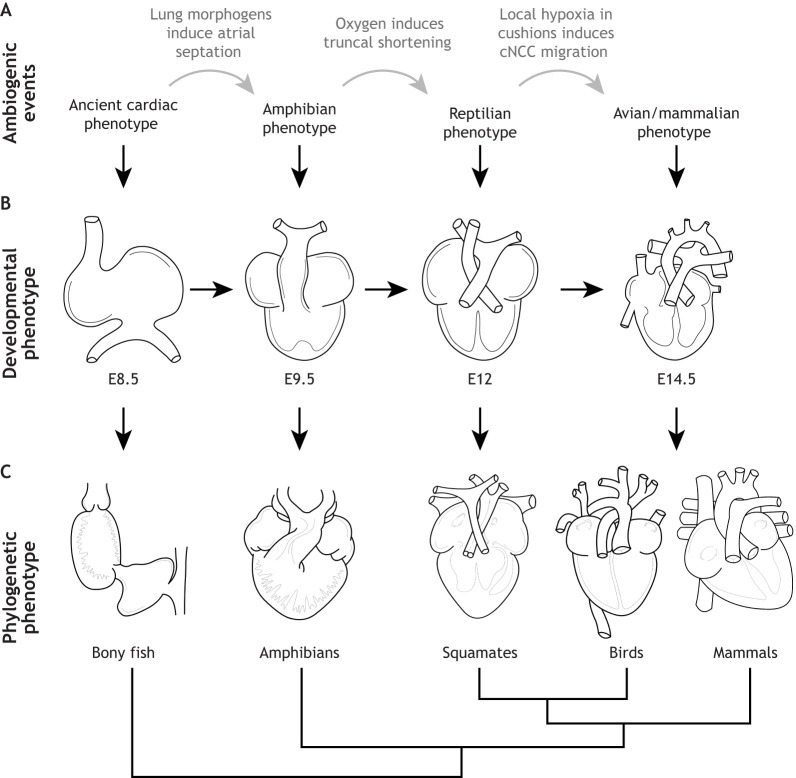
**Normal mammalian cardiogenesis and ambiogenic events hypothesized to be required for proper outflow tract development during placentation, along with associated developmental and phylogenetic phenotypes.** (A) Mammalian heart morphology at different times in development, along with the ambiogenic event potentially required to bring about the next morphological change. (B) Associated mouse cardiac phenotypes at different developmental stages. (C) According to the EDDI model, the genetically fixed developmental program underlying cardiogenesis produces the coiled heart tube as seen in bony fish. If lungs are present, atrial septation is mediated by molecular signals secreted from the developing lungs, which leads to the development of an ‘amphibian’ phenotype. Oxygenation of cardiac tissue via the onset of placental respiration induces shortening of the TA so that the aortic arches directly contact the ventricle, as is seen in the ‘reptilian’ phenotype. Intricate interactions between mechanical stressors and local oxygen environments then coordinate the migration of cNCCs into the cardiac cushions, where they complete the aorticopulmonary septum. This leads to the formation of a functional double circulation. Abbreviations as in Figs 1 and 3.

Disruptions in the pathways regulating these processes can lead to congenital heart disease (CHD; Figs 3 and 4; Kelly et al., 2014). Although genetic mutations have been identified in some familial and spontaneous cases of CHD, the majority of cases cannot be attributed to a single genetic cause (Fahed et al., 2013). However, non-genetic factors, such as placental abnormalities (Burton and Jauniaux, 2018; Kalisch-Smith et al., 2024; Nijman et al., 2024), high-altitude living (Hasan, 2016; Miao et al., 1988; Song et al., 2021), smoking (Lee and Lupo, 2013; Wang et al., 2022; Zhang et al., 2017) and prenatal diabetes (Basu and Garg, 2018; Choudhury et al., 2021; He et al., 2024; Ibrahim et al., 2023; Morishima et al., 1996; Zhao et al., 2020; Zhao, 2010), have been associated with an increased risk of CHD, though the exact mechanisms remain unclear (Dunwoodie, 2009; Giussani, 2021; Patterson and Zhang, 2010; Kenchegowda et al., 2014; Patel and Burns, 2013; Simeone et al., 2016; Webster and Abela, 2007).

Notably, most CHDs in humans involve disruptions in the terminal stages of the SHF contribution to the heart, particularly affecting OFT formation. This process, which transforms the heart's single OFT into a separated pulmonary artery and aorta, is frequently disrupted, leading to malformations of the double-circulatory system (Fisher and Burggren, 2007; Gittenberger-de Groot et al., 2005; Pierpont et al., 2018; Zaidi and Brueckner, 2017).

I propose that the variability of the OFT and its susceptibility to malformation both stem from temporal differences in progenitor cell differentiation. Early cardiogenesis, and thus early progenitor differentiation, occurs under hypoxic conditions for all vertebrate species. But SHF differentiation, which is critical for OFT septation, coincides with species-specific changes in oxygen availability linked to developmental mode (e.g. placentation, timing of egg deposition and CAM formation). Oxygen levels influence proliferation and differentiation of multipotent progenitor cells (Fisher and Burggren, 2007; Gittenberger-de Groot et al., 2005), and shifts in oxygen regime could therefore shape OFT development, leading to lineage-specific phenotypes (Figs 3 and 4). Additionally, altered oxygen levels are frequently associated with cardiac malformations (Dunwoodie, 2009; Giussani, 2021; Patterson and Zhang, 2010; Kenchegowda et al., 2014; Patel and Burns, 2013; Simeone et al., 2016; Webster and Abela, 2007).

This view contrasts with the idea that the basic cardiac blueprint was expanded during evolution by genetically assimilating pre-existing developmental pathways into the core program of cardiogenesis. In the ‘genetic assimilation’ model, mutations in the expanded cardiac program are thought to underlie CHD (Olson, 2006; Taussig, 1988). However, the high number of CHD cases with unknown causes and the numerous external factors that can disrupt normal cardiogenesis suggest that formation of these additional cardiac structures may not be driven by genetic mutations in the core heart development program. Below, I discuss major events in cardiogenesis and outline how certain developmental abnormalities correspond with the cardiac phenotypes we see in phylogeny.

#### Oxygen delivery to the embryo and embryonic heart

The hearts of developing anamniotes (fishes and amphibians) pump oxygen-poor blood to the gills, where it is oxygenated and dispersed to supply all organs. Thus, the embryo receives enough oxygen for overall development but the cardiac tissue contacts oxygen-depleted blood during critical developmental stages (Fig. 1; Kardong, 2019; Kraus and Metscher, 2022). In contrast, the hearts of amniotes (squamates, crocodilians, birds and mammals) receive oxygenated blood during development, owing to different strategies of embryonic gas exchange (Fig. 1). In reptiles, crocodilians and birds, oxygen diffuses into the blood through the eggshell. As the CAM forms and expands, the surface area for gas exchange increases further to keep up with an increasing metabolic demand of the growing embryo (Schlatter et al., 1997).

In mammals, placental respiration begins once the embryonic circulation is fully closed. This is achieved when the developing placenta invades the maternal uterine spiral arteries. Initially, blood flow from these arteries to the embryo is blocked by endovascular plugs. In humans, small channels within these plugs begin to form around 6–7 weeks of gestation (Carnegie stages 10–17; see Glossary; note that Carnegie stages are counted from fertilization, whereas pregnancy is usually dated from the last menstrual period, creating a ∼2-week offset). Over time, the plugs gradually disintegrate, allowing continuous blood flow by 8–10 weeks of gestation (Carnegie stages 18–23; Pijnenborg et al., 2006; Roberts et al., 2017; Sato, 2020; Faber et al., 2021). This transition coincides with the heart's shift from a simple, unidirectional tube to a structurally developed organ at the end of embryonic development. A similar time line occurs in mice, despite the species being at otherwise different stages of development: as fetal–maternal blood flow starts at around embryonic day (E)9.5, the heart undergoes abrupt developmental changes (Figs 3 and 4; Jauniaux et al., 2000; Graham et al., 2015; Buijtendijk et al., 2020; Hu and Zhang, 2021). Although oxygen saturation in mammals remains low compared with that experienced during postnatal life, disintegration of the spiral artery plugs increases oxygen availability from about 2.5% to 8% during the first trimester (Morton and Brodsky, 2016; Saghian et al., 2019; Burton et al., 2021).

### Apoptosis and OFT remodeling

In mammals, birds and crocodilians, OFT remodeling involves the incorporation of the conus arteriosus into the ventricle, proper alignment of the OFT with the ventricles and the shortening of the truncus arteriosus through programmed cell death (i.e. apoptosis; see Fig. 3 for a depiction of the conus arteriosus and truncus arteriosus; Gittenberger-de Groot et al., 2005; Barbosky et al., 2006). Previous studies suggest that tissue hypoxia induces this apoptosis (Anderson et al., 2003; Barbosky et al., 2006; Rothenberg et al., 2003; Sugishita et al., 2004a) through hypoxia-inducible factor (HIF), which upregulates the main biochemical pathway for gene-based defenses against oxygen deprivation (Fisher and Burggren, 2007; Semenza, 1998, 1999). Forced expression of *HIF-1a* has been shown to protect cardiomyocytes from apoptosis during OFT remodeling, actually causing OFT defects (Sallee et al., 2004; Sugishita et al., 2004a,b,c). This suggests that the presence of oxygen-rich blood and consequent HIF degradation potentially triggers OFT remodeling (Figs 3 and 4).

In fishes and amphibians, the conus arteriosus does not integrate into the ventricle, and the truncus arteriosus does not shorten by apoptosis during OFT development (Fig. 1; Wang et al., 2018; Park et al., 2023). As their developing hearts contact oxygen-poor blood, *HIF* expression is likely to be upregulated, as seen in *Xenopus laevis* (Nagao et al., 2008). Taken together, this suggests that when the OFT contacts oxygen-poor blood, HIF maintains its downstream cell-protective effects, hindering apoptosis-driven OFT shortening. Once oxygenated blood flows through the heart, *HIF* downregulation triggers apoptosis and OFT remodeling, as seen in amniotes (Fig. 3; Sugishita et al., 2004a,b,c; Barbosky et al., 2006; Liu and Fisher, 2008).

If the above predictions are correct, then restricting oxygen during the apoptotic window in development of mammals, birds or crocodilians should inhibit correct OFT development (Watanabe et al., 1998). Indeed, our lab was able to experimentally confirm this prediction in chick embryos (Kraus et al., 2025 preprint).

### Cardiac cushions

Vascular endothelial growth factor (VEGF), a family of growth factors that stimulate angiogenesis, is downstream of HIF. As *HIF* expression is downregulated in the presence of oxygen, so is *VEGF* expression. Although the downregulation of *VEGF* is necessary to initiate apoptosis and OFT remodeling, complete inhibition of *VEGF* can lead to heart defects in itself (Sugishita et al., 2004a,b,c). This can potentially be explained by variations in local oxygen availability within the developing heart. In early mammalian and avian development, the endocardium and myocardium are separated by an extracellular matrix that is thin enough to allow oxygen diffusion to the myocardium (Gittenberger-de Groot et al., 2005; Sugishita et al., 2004b,c). As development progresses, endocardial cells lining the inside of the heart undergo an endothelial-to-mesenchymal transition (EndoMT; see Glossary) and migrate into the extracellular matrix. The resulting mesenchymal cells proliferate and form thick cardiac cushions, or heart valve progenitors, between the myocardium and the endocardium. As a result, oxygen no longer diffuses all the way to the myocardium, causing local hypoxia in the myocardium beneath the cushions, with consequential local upregulation of *HIF* and subsequent *VEGF* expression (Fig. 3; Camenisch et al., 2010, 2000; Djenoune et al., 2022; Garside et al., 2013; Runyan et al., 1990). *VEGF* expression is needed locally for proper cardiac cushion remodeling, as EndoMT is regulated by *VEGF* in a dose-dependent manner (Lambrechts and Carmeliet, 2004). Alterations in both global and local OFT tissue oxygenation can thus lead to conotruncal heart defects (see Glossary), providing a good example of ambiogenic induction (Fig. 3).

#### Neural crest cells

Neural crest cells are a vertebrate-specific innovation. They originate from the developing central nervous system, and migrate extensively via the aortic arches to produce a plethora of cell lineages (Bronner and LeDouarin, 2012). A subpopulation of neural crest cells, the cardiac neural crest cells (cNCCs), migrates towards the heart. In amniotes, cNCCs migrate deep into the distal OFT, where they differentiate into smooth muscle cells and connect the arterial arches with the ventricular septum, thereby forming a complete aorticopulmonary septum. In anamniotes, cNCCs migrate towards the OFT but remain in the pharyngeal arches. Thus, amniotes either completely lack an aorticopulmonary septum or have an incomplete one, as is often seen in squamates (Figs 3 and 4; Hutson and Kirby, 2003; Abu-Issa et al., 2004; Kulesa et al., 2010).

In humans, failure of cNCC migration can result in an absent aorticopulmonary septum, causing congenital heart defects (Poelmann et al., 2017)*.* This can be seen in DiGeorge syndrome, a chromosomal disorder where one copy of the *Tbx1* gene is absent (Burn et al., 1993). Affected individuals show cardiovascular abnormalities characterized by an incomplete aorticopulmonary septum (Théveniau-Ruissy et al., 2008). *Tbx1* has been found to upregulate *Fgf8*, which inhibits *Sema3C*, a chemoattractant that guides cNCCs into the OFT (Barriga et al., 2013; Kodo et al., 2017; Neeb et al., 2013; Toyofuku et al., 2008; Xu et al., 2004). *Fgf8* also fails to be upregulated under hypoxic conditions, phenocopying defects caused by *Tbx1* deletion (Fig. 3; Gandhi et al., 2020).

**Fig. 5.**
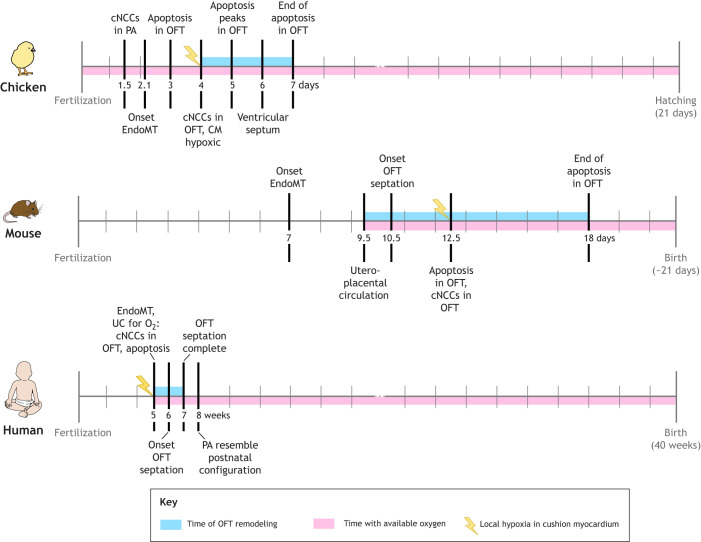
**Developmental time line of outflow tract remodeling during ontogeny of chick, mouse and human, with corresponding environmental conditions.** Independent of the order of individual events and external triggers, the EDDI model predicts that all precursors needed for the transition to a double circulation need to be present to initiate OFT remodeling. Once the heart receives oxygenated blood, apoptosis sets in. However, it is only if the EndoMT has proceeded far enough to produce hypoxia in the cushion myocardium that cNCCs start migrating into the OFT, culminating in drastic OFT remodeling. Note the differences in time scale between different species. (Information taken from Al Naieb et al., 2013; Buijtendijk et al., 2020; Graham et al., 2015; Hamburger and Hamilton, 1951; Hikspoors et al., 2022; O'Rahilly and Müller, 2010.) CM, cushion myocardium; PA, pharyngeal arches; UC, umbilical cord; other abbreviations as in Figs 1 and 3.

Taken together, the above discussion suggests that the oxygenated OFT of amniotes is likely to inhibit cNCC migration. However, as described above, hypoxia occurs in the myocardium underlying the OFT cushions (Liu and Fisher, 2008). It is possible that this local hypoxia downregulates the *Tbx1* target *Fgf8* in the cushion area and thus locally upregulates *Sema3C*. *Sema3C* expression might then prompt a subset of cNCCs to migrate from the pharyngeal arches into the distal OFT (Fig. 3). This idea is supported by *Sema3C* expression observed in the developing mouse OFT at E10.5, coinciding with endocardial cells populating the cushions (Keyte and Hutson, 2012; Keyte et al., 2014). In avian species, cNCCs reach the pharyngeal arches around Hamburger–Hamilton stage (HH)11 (see Glossary), before EndoMT initiation at HH16. However, these cells do not migrate into the OFT until about HH21, despite the OFT being relatively normoxic earlier than in mammals. This suggests a threshold of hypoxia must be crossed in the cushions to downregulate *Fgf8* and upregulate *Sema3C* (Hamburger and Hamilton, 1951; Hutson and Kirby, 2003; Kirby et al., 1983; Kodo et al., 2017; Martinsen, 2005). This mechanism fits with ambiogenic induction and EDDI, as all necessary factors must converge to induce certain changes to the core developmental program, even if the sequence of events differs between birds, crocodilians and mammals (Figs 3, 4 and 5).

#### Mechanical forces

In addition to hypoxia, mechanical forces such as shear stress further regulate cNCC behavior and OFT development through *Klf-2* and *TGF-β* signaling pathways. Shear forces, combined with oxygen gradients, create a multifactorial environment that ensures precise OFT remodeling in amniotes (Fig. 3; Hu and Clark, 1989; Liu et al., 2009; Vermot et al., 2009; Yalcin et al., 2011; ten Dijke et al., 2012).

The endocardium lining the inside of the heart consists of a single layer of endothelial cells, each of which is equipped with a mechanosensitive cilium. In normal cardiogenesis, shear stress bends this cilium, which causes *Klf-2* expression. When *Klf-2* is expressed, endothelial cells maintain a quiescent, anti-inflammatory phenotype. *Klf-2* is also needed to regulate the deposition of matrix proteins in the cardiac cushions and has important downstream effects on *Wnt* and *BMP4*, both of which are needed for correct OFT development (Combs and Yutzey, 2009; Garside et al., 2013; Luxán et al., 2016; Potts and Runyan, 1989). As the cardiac cushions expand during EndoMT, shear forces continually increase in the cushion area, eventually causing the cilia to break away from endothelial cells exposed to higher levels of shear stress. De-ciliated endothelial cells are no longer able to express *Klf-2*, which triggers *TGF-β* signaling, leading to a proinflammatory phenotype (Boon et al., 2007; Egorova et al., 2012, 2011a,b). *Klf-2* expression in still-ciliated cells is needed to control essential downstream targets of *TGF-β* while simultaneously upregulating *Notch* signaling. Downregulation of *Notch* has been shown to downregulate *Fgf8*; thus, shear stress is predicted to modulate cNCC behavior through *Klf-2* and *TGF-β*. Additionally, *TGF-β* controls the expression of *Foxc1* and *2*, which have been shown to positively regulate *Sema3c* expression (Duchemin et al., 2019; Goddard et al., 2017; Inman et al., 2018; Seo and Kume, 2006). Indeed, the expression of *TGF-β* and that of its downstream targets *Foxc1* and *2* spatiotemporally coincides with *Sema3c* expression and cNCC migration in amniotes. Thus, shear stress is another potential trigger for ambiogenic induction (Fig. 3; High and Epstein, 2008; Keyte et al., 2014; Neeb et al., 2013).

#### A note on squamates and aortic arches

The predictions outlined above suggest that, in squamates, developmental oxygenation is sufficient to downregulate global HIF expression in the OFT, leading to apoptosis-driven shortening of the truncus arteriosus. Uniquely, squamates are the only order in which this stage of OFT remodeling occurs without subsequent migration of cNCCs into the distal OFT to complete the aorticopulmonary septum.

In birds, the left visceral aorta, derived from pharyngeal aortic arch 4 (PAA4), regresses during development, whereas the right visceral aorta persists and merges with cNCC-derived elements to form the aorticopulmonary septum. In mammals, by contrast, both PAA4-derived structures persist, but the right PAA6 regresses, whereas the left PAA6 contributes to the septum. Thus, the aorticopulmonary septum in birds incorporates cNCC- and SHF-derived cells associated with PAA4, whereas in mammals it incorporates cNCC- and SHF-derived cells from PAA6 (see Poelmann et al., 2017, for a detailed discussion).

Squamates differ in that the left visceral aorta is positioned slightly differently than in birds and crocodilians. Rather than regressing during development, it persists and appears to act as a barrier that separates cNCC and SHF populations (Poelmann et al., 2017). This arrangement is likely to produce shear stress patterns that are distinct from those of birds and crocodilians. As discussed above, such differences in hemodynamics can influence EndoMT directly through the *Klf-2*/*TGF-β* pathway, and indirectly by modifying the extracellular matrix composition of the cardiac cushions. Consistent with this, changes in the expression of *Fibulin-1* (a matrix protein) have been shown to alter cNCC migration and apoptosis (Cooley et al., 2008), while altered *TGF-β* expression is associated with changes in apoptosis and with arch artery abnormalities in mammals (Molin et al., 2002). Although definitive data are currently lacking, this again emphasizes how different environmental cues might influence and coordinate development.

### Mechanisms and evolutionary consequences of EDDI in heart development

According to the EDDI model proposed here, developmental changes might be regulated by environmental conditions such as hypoxia, which modulate gene expression to guide morphogenesis. This would allow for species-specific variation in cardiogenesis, influenced by local factors such as oxygen availability. Under this model, in placental mammals, the onset of placental oxygenation triggers the degradation of HIF, altering the interaction between interdependent parts of the cardiac developmental program, including cushion formation, EndoMT and shear response. These changes affect localized conditions, such as hypoxia in the cushion myocardium, which attracts cNCCs and incorporates additional developmental networks into cardiogenesis. Ultimately, these factors shape cell-fate decisions, altering how cardiac progenitors differentiate.

EDDI provides a potential explanation for why certain congenital heart defects in humans resemble normal phenotypes in other species. As the embryo develops and encounters environmental cues, ambiogenic induction expands the genetic toolkit available for the cardiac developmental program, allowing new regulatory patterns to emerge and new cardiac structures to form. For example, persistent truncus arteriosus in humans, which presents as a single arterial trunk (unshortened truncus arteriosus) with malalignment-type ventricular septal defects, resembles the normal OFT development in amphibians and lungfish, where low oxygen and the absence of apoptosis are part of standard development. The EDDI model suggests that one potential reason for this condition in humans is insufficient oxygenation at the onset of placental respiration, which limits apoptosis and restricts further developmental expansion in the heart (Figs 3, 4 and 5).

Oxygen deprivation at other points in development might not block apoptosis but could still bias development, affecting processes such as cushion formation and cNCC migration. In these cases, the EDDI model predicts milder cardiac malformations resembling a ‘reptilian’ phenotype, where the OFT partially divides but lacks a complete aorticopulmonary septum. Therefore, EDDI offers an alternative to Haeckelian explanations (see Glossary) for why some cardiac defects mimic ancestral vertebrate phenotypes. When key environmental triggers – such as sufficient oxygen during OFT apoptosis – are absent, cardiogenesis might revert to its core developmental processes, a process known as atavism (see Glossary; Figs 3 and 4). This view aligns with the observation that tissues evolve similar morphologies under comparable environmental pressures (Loffet et al., 2023), supporting Garstang's (1922) famous conclusion: ‘Ontogeny does not recapitulate phylogeny, it creates it’.

## Conclusions and future directions

The concept of using ontogenetic rules to understand trait evolution is not novel, but applying this framework to cardiogenesis and congenital heart disease can offer new insights. My exploration of this idea led to the hypothesis that the cardiac developmental program has not fully genetically assimilated the co-opted sub-circuits needed to modify cell-fate decisions and create accessory structures in response to increased metabolic demands. Instead, I propose that the expansion of the core developmental program is driven by ambiogenic induction, where environmental triggers initiate physiological responses that expand the genetic toolkit available to cardiac progenitor cells. These newly activated elements interact with the core developmental program, leading to the formation of new cardiac structures through coordinated environmental and genetic interactions (Figs 3, 4 and 5).

This model builds on prior frameworks that emphasize phenotypic plasticity, but it diverges in a key way: although earlier models require genetic assimilation to stabilize new phenotypes across generations (Baldwin, 1896; Levis and Pfennig, 2016; Wagner et al., 2019; West-Eberhard, 2005), EDDI does not depend on such assimilation. By offering a mechanism in which environmental cues play a primary role, EDDI provides a fresh perspective on why the heart remains vulnerable to environmental perturbations throughout its evolutionary history. Moreover, EDDI might broaden our understanding of congenital heart disease in cases without clear genetic defects, while also helping to clarify how development might be disrupted in genetically based heart defects, such as those associated with DiGeorge syndrome.

As the heart is the first organ to form and function, its development is not dependent on that of other organ systems, making it comparatively easy to tease apart different (micro-)environmental factors. Thus, the heart serves as an ideal model to unravel some of the intricate interactions governing development. As cardiogenesis is vulnerable to environmental perturbation, it exemplifies how developmental processes are sensitive to non-genetic signals, such as oxygen levels and shear stress.

Although this work focuses on how specifically oxygen and shear stress affect vertebrate heart development, the principles of EDDI could be extended to other organ systems and environmental factors (for examples, see Gilbert, 2012; Miyakawa et al., 2010). Ambiogenic induction might also be crucial in guiding species-specific limb development in vertebrates (Newman and Müller, 2005).

The EDDI model proposed here potentially offers a new framework for generating testable hypotheses for various fields of study, particularly in the light of new methods such as single-cell -omics and metabolomics, and gene–environment interaction studies. Considering development through the lens of the EDDI model might thus reveal broader patterns of environmentally driven development, providing a deeper understanding of evolution across biological systems.
